# Supplementary material for: Mathematical Modeling of Oncolytic Virus Therapy Reveals Role of the Immune Response
Source: Viruses. 2023 Aug 25;15(9):1812. doi: 10.3390/v15091812 (PMC10536413; doi:10.3390/v15091812)
Supplement: Supplementary file 1 [file viruses-15-01812-s001.zip › viruses-2524274-supplementary.pdf]

# Mathematical Modeling of Oncolytic Virus Therapy Reveals Role of the Immune Response: Supplementary Documents

Ela Guo, Hana M. Dobrovolny

July 1, 2023

## 1 Basic reproductive number

The basic reproductive number, $R_0$, was calculated based off the work of Diekmann et. al. using Next Generation Matrices (NGM) [1].

Transmission Matrix (T)

$$\begin{pmatrix} 0 & 0 & \beta T_0 \\ 0 & 0 & 0 \\ 0 & 0 & 0 \end{pmatrix}$$

Transition Matrix ($\Sigma$)

$$\begin{pmatrix} -k & 0 & 0 \\ 0 & -\delta & 0 \\ 0 & \frac{p}{1+\epsilon F} & -c \end{pmatrix}$$

$-T\Sigma^{-1} =$

$$\begin{pmatrix} 0 & \frac{\beta T p}{\delta c(1+\epsilon F)} & \frac{\beta T}{c} \\ 0 & 0 & 0 \\ 0 & 0 & 0 \end{pmatrix}$$

Dominant eigenvalue of $-T\Sigma^{-1}$: $\frac{\beta T p}{\delta c(1+\epsilon F)}$

## 2 Corner Plots

The following plots show correlation relationships between the parameters, as well as parameter histograms. Plots were made using the `corner.py` package of Python [2].

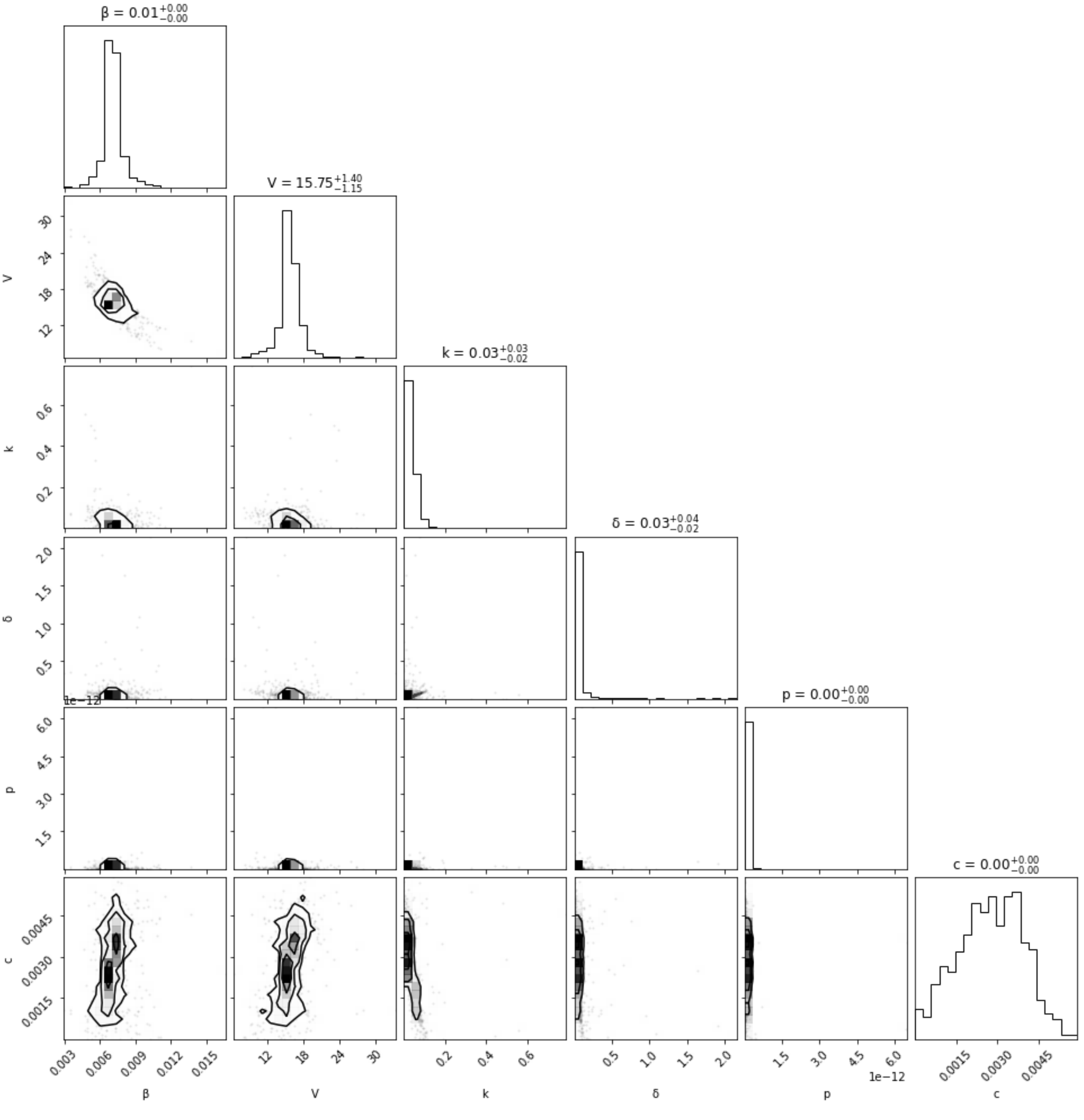


Figure 1: Ad1d24.P19 no immune response.

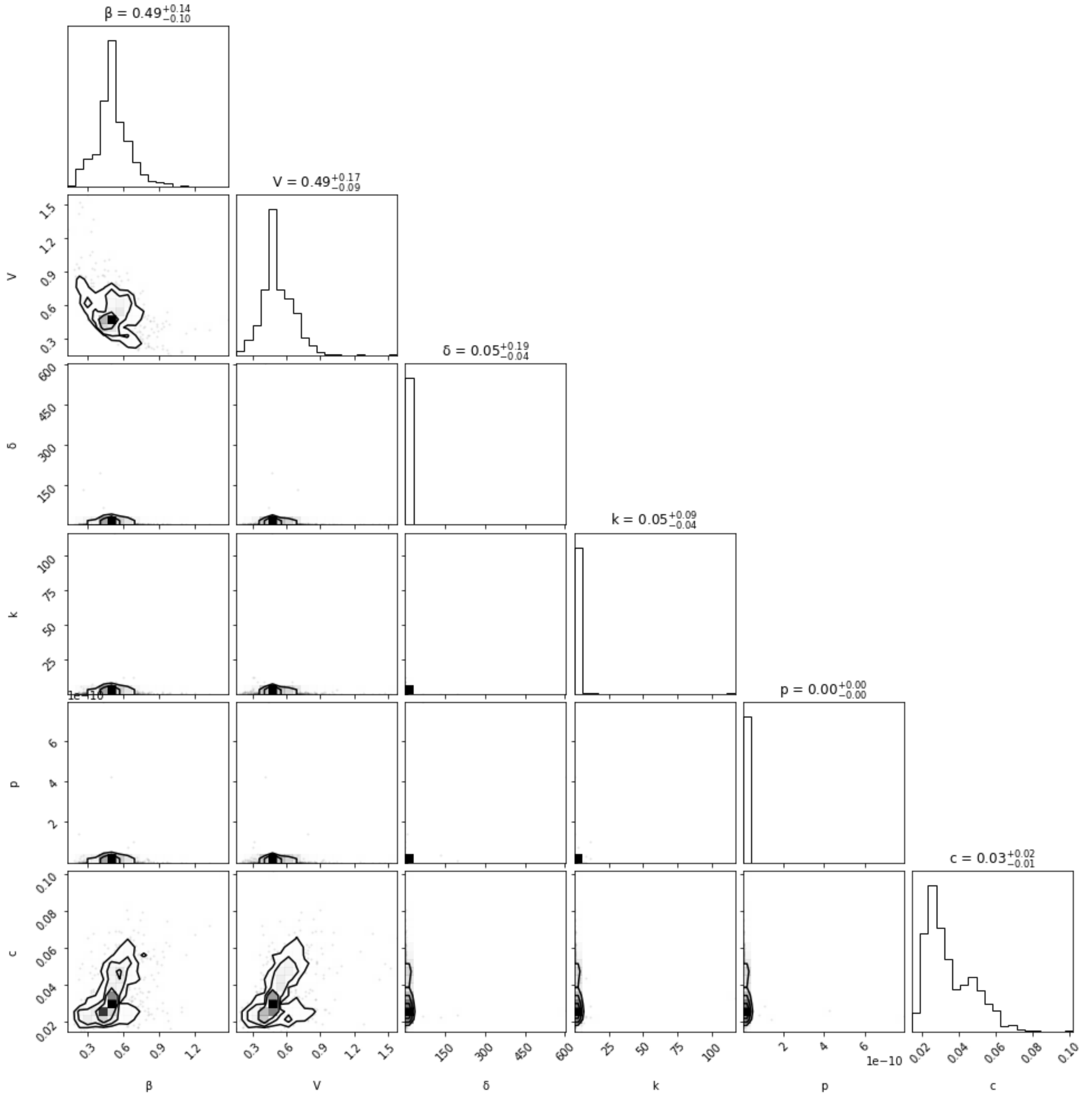


Figure 2: Ad2d24.P19 no immune response.

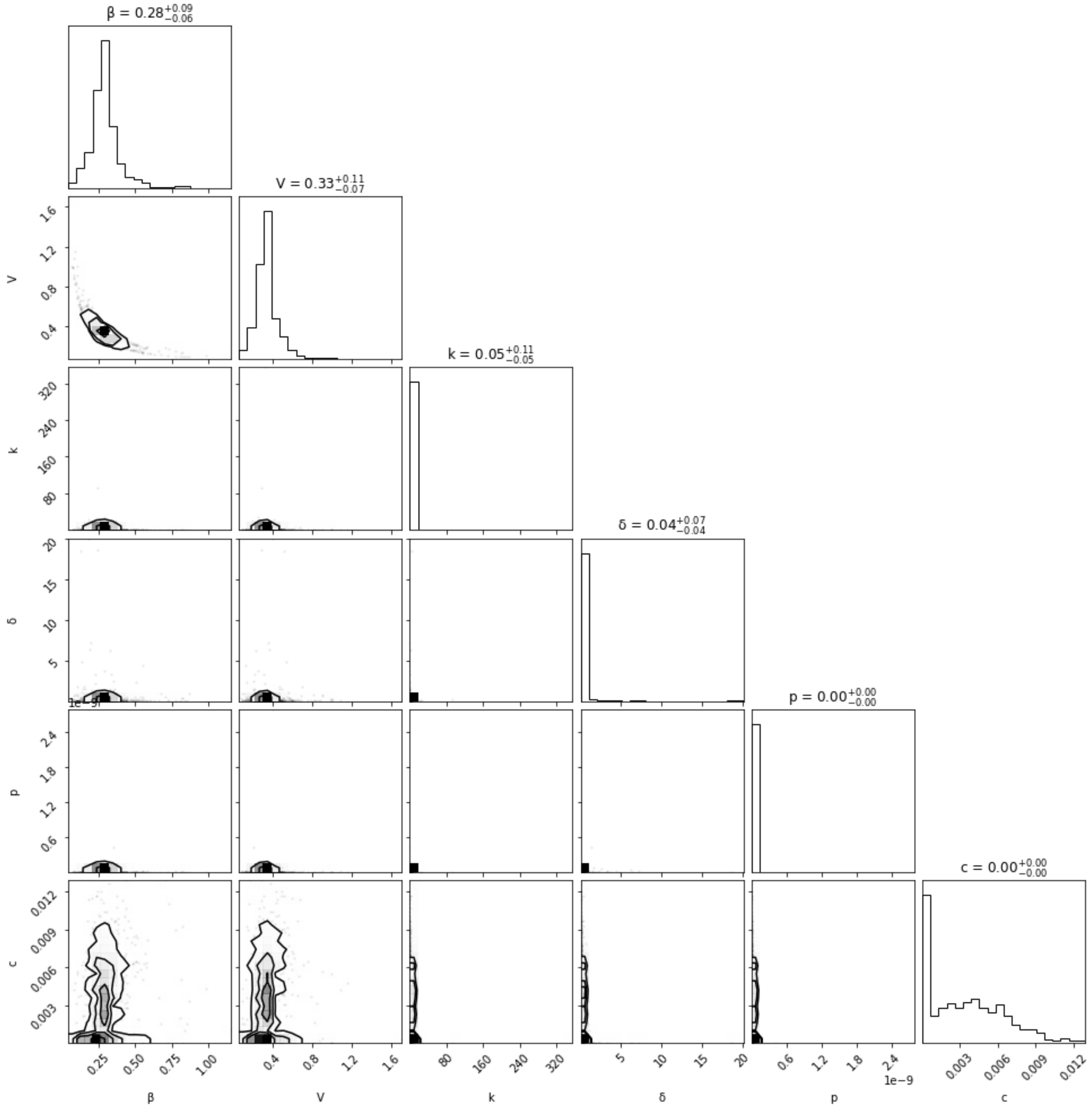


Figure 3: Ad5d24.P19 no immune response.

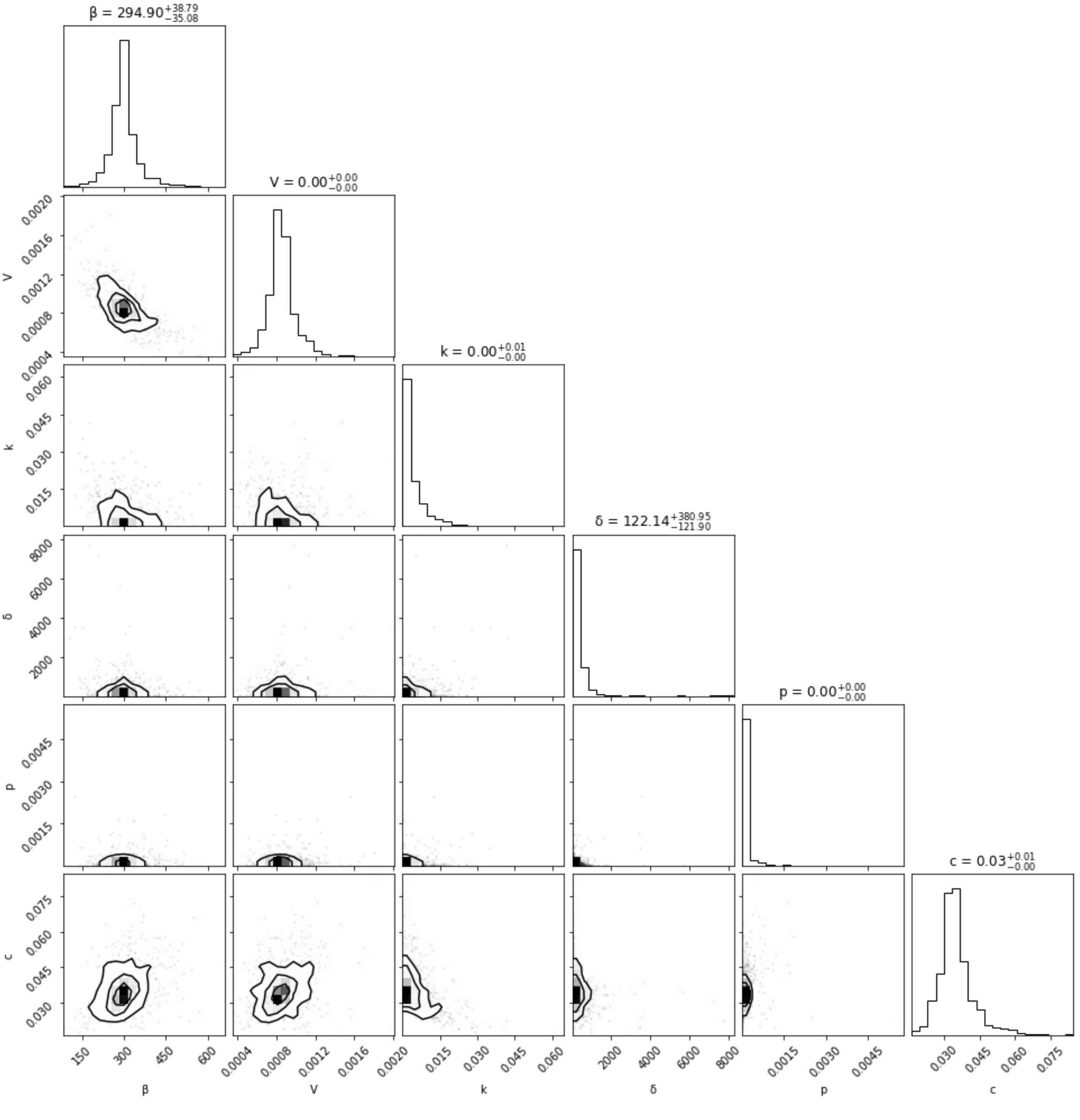


Figure 4: Ad6d24.P19 no immune response.

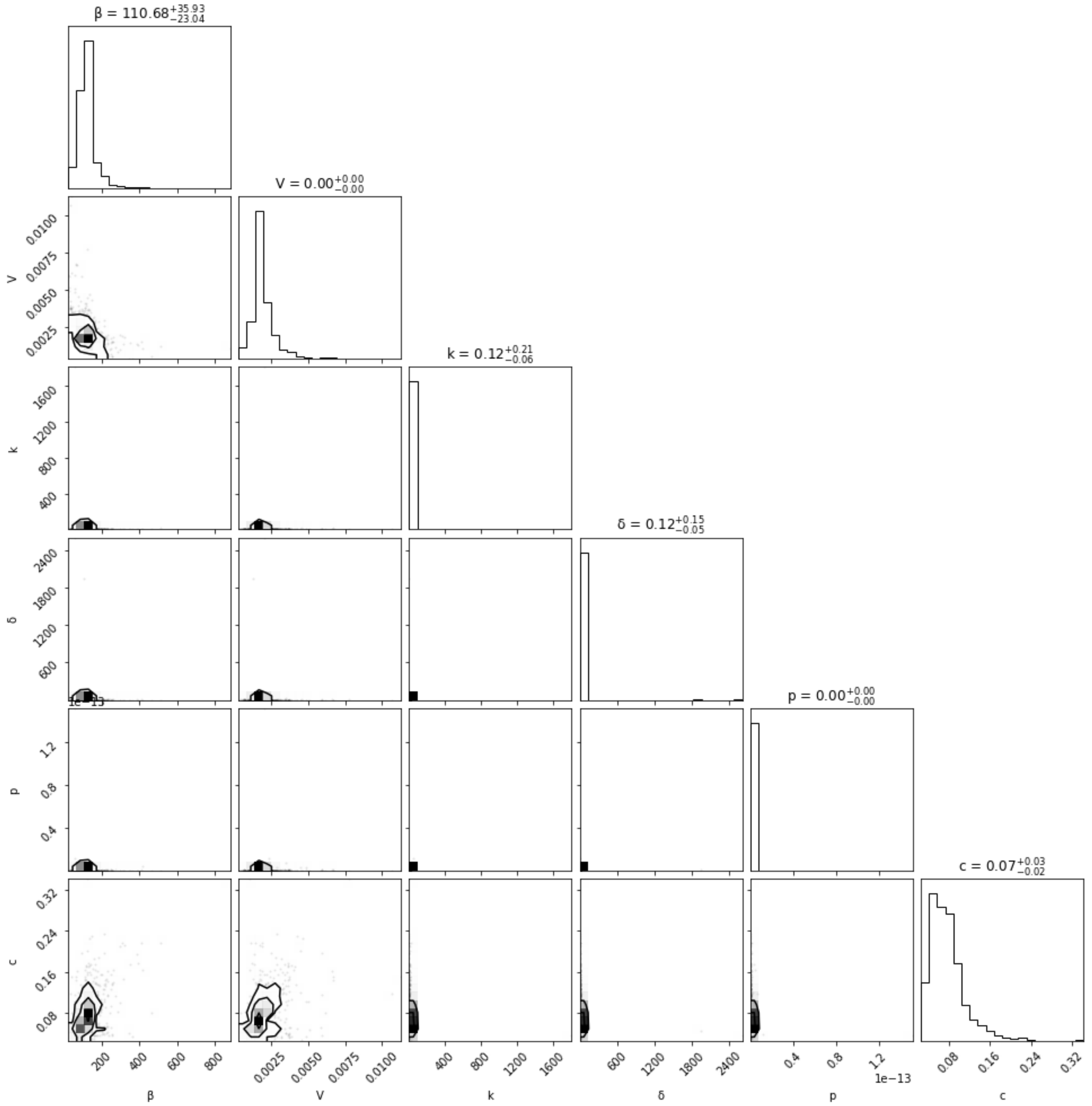


Figure 5: H101 no immune response.

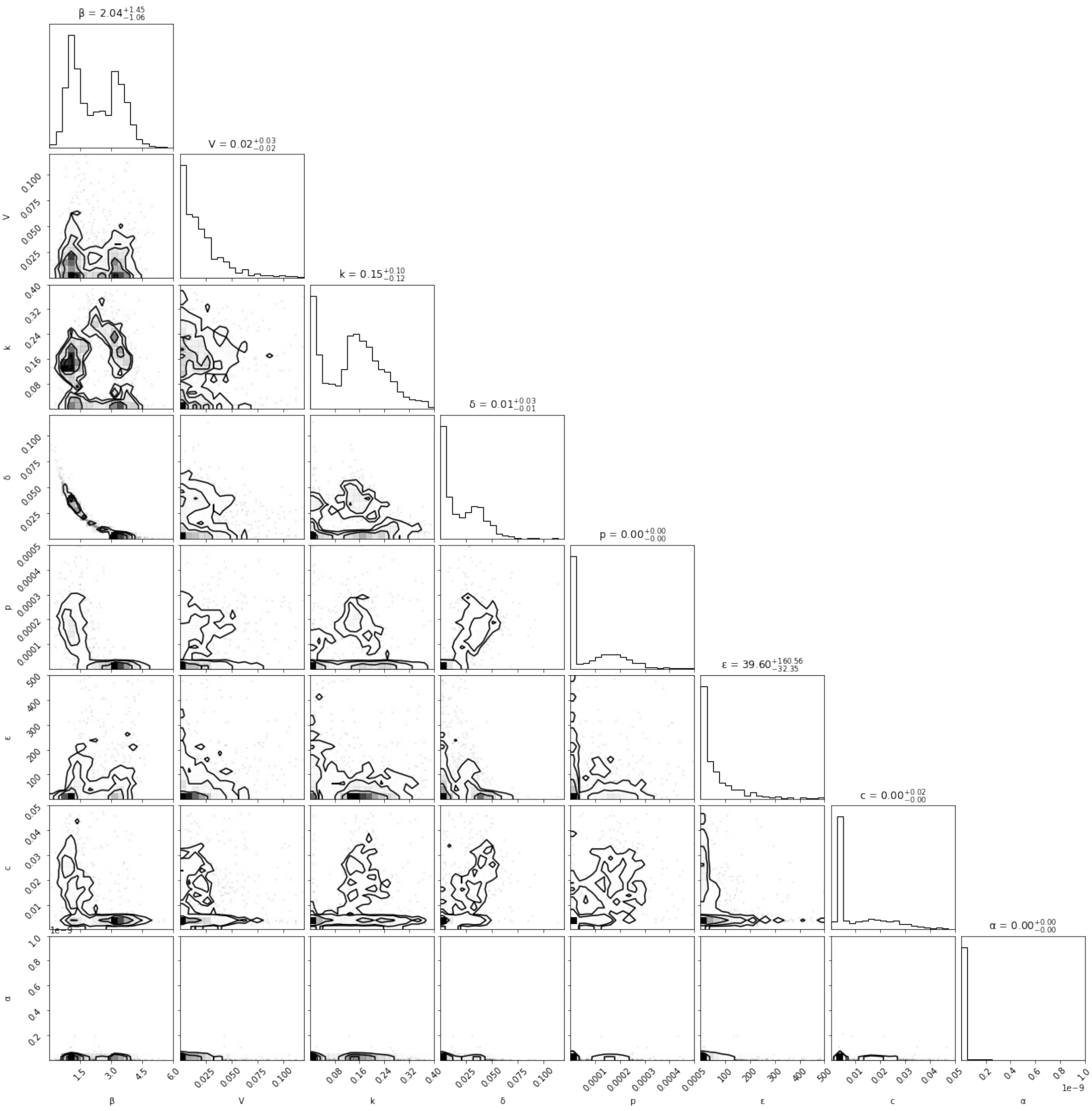


Figure 6: Ad1d24.P19 with immune response.

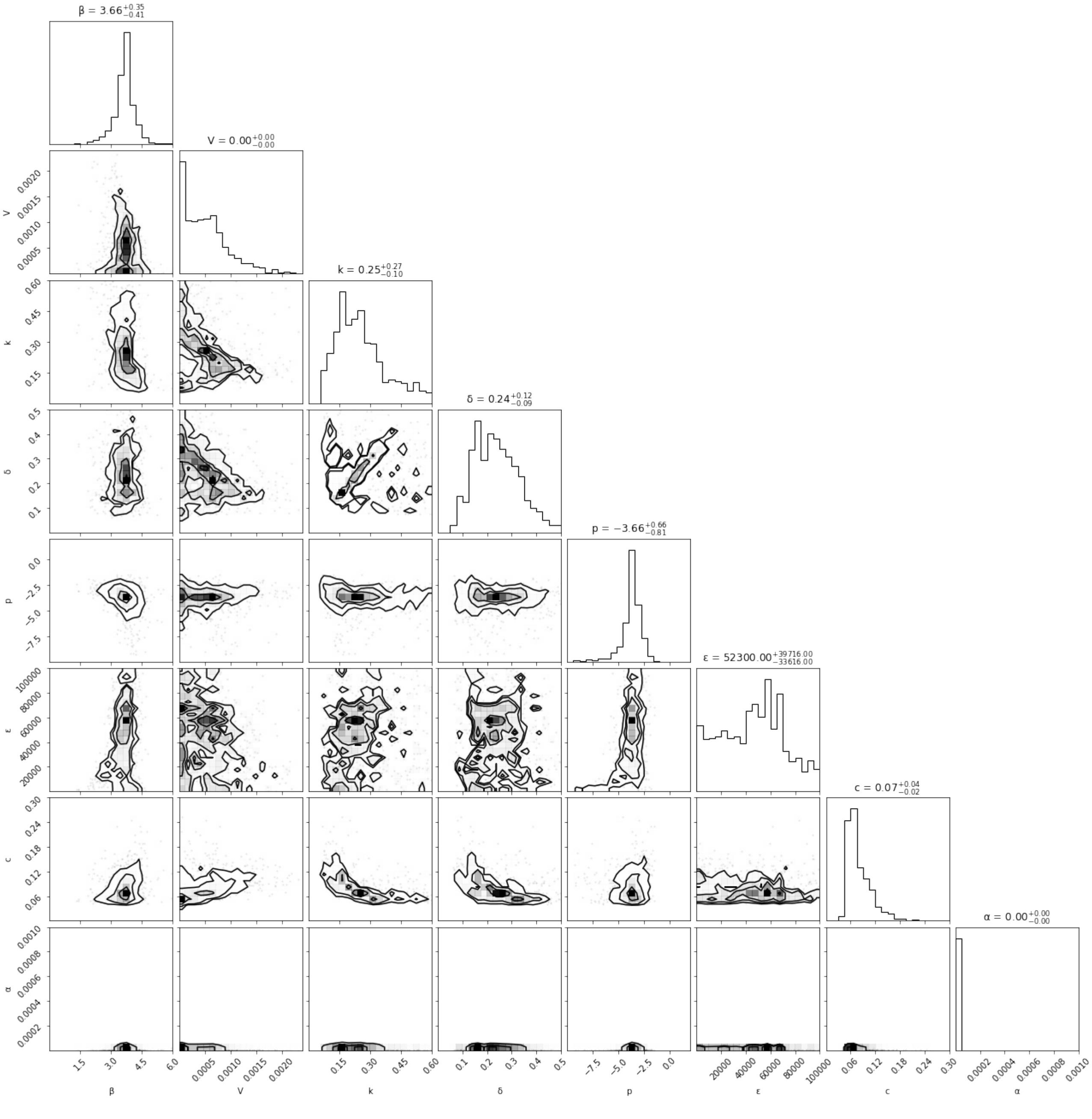


Figure 7: Ad2d24.P19 with immune response.

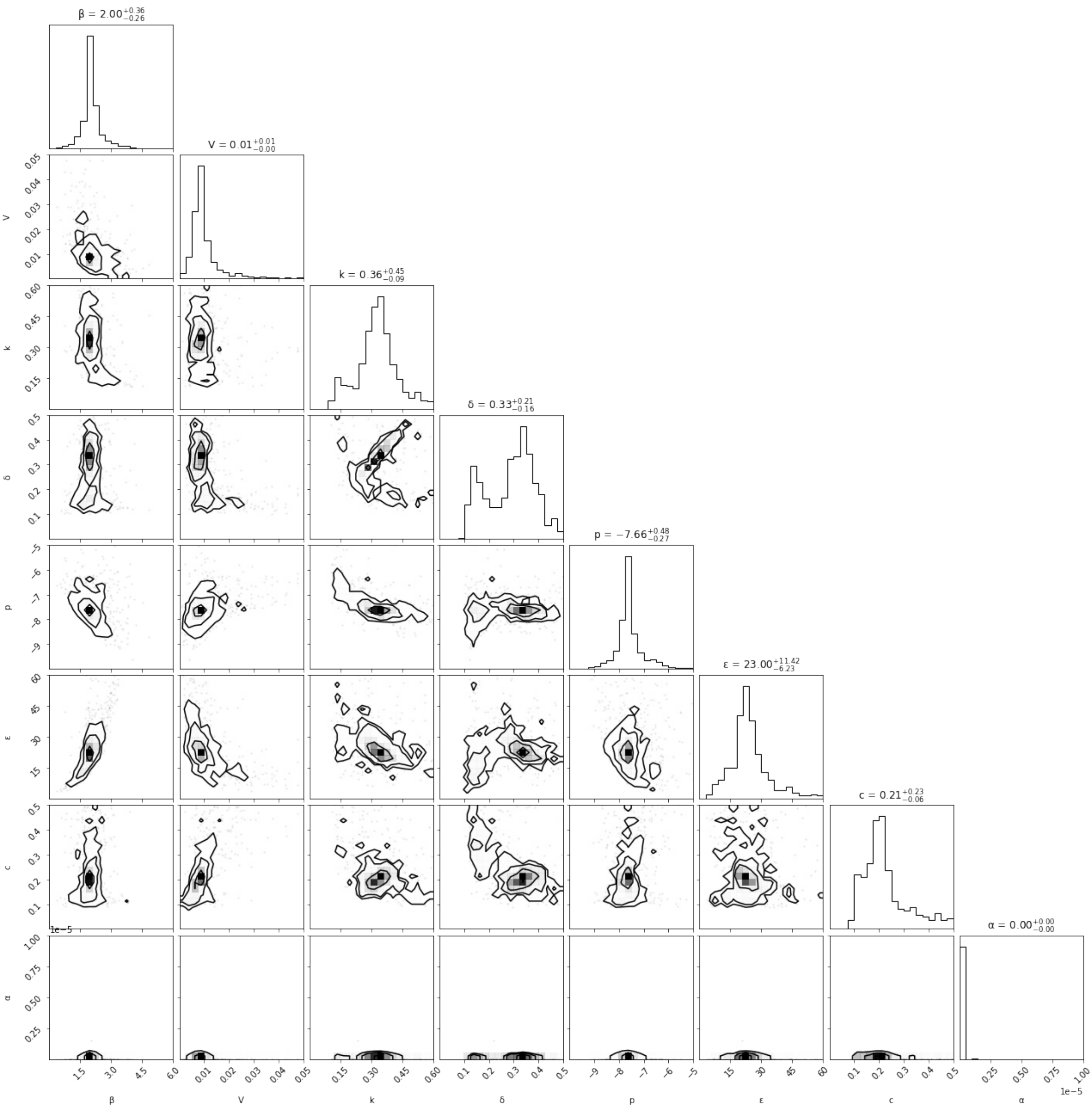


Figure 8: Ad5d24.P19 with immune response.

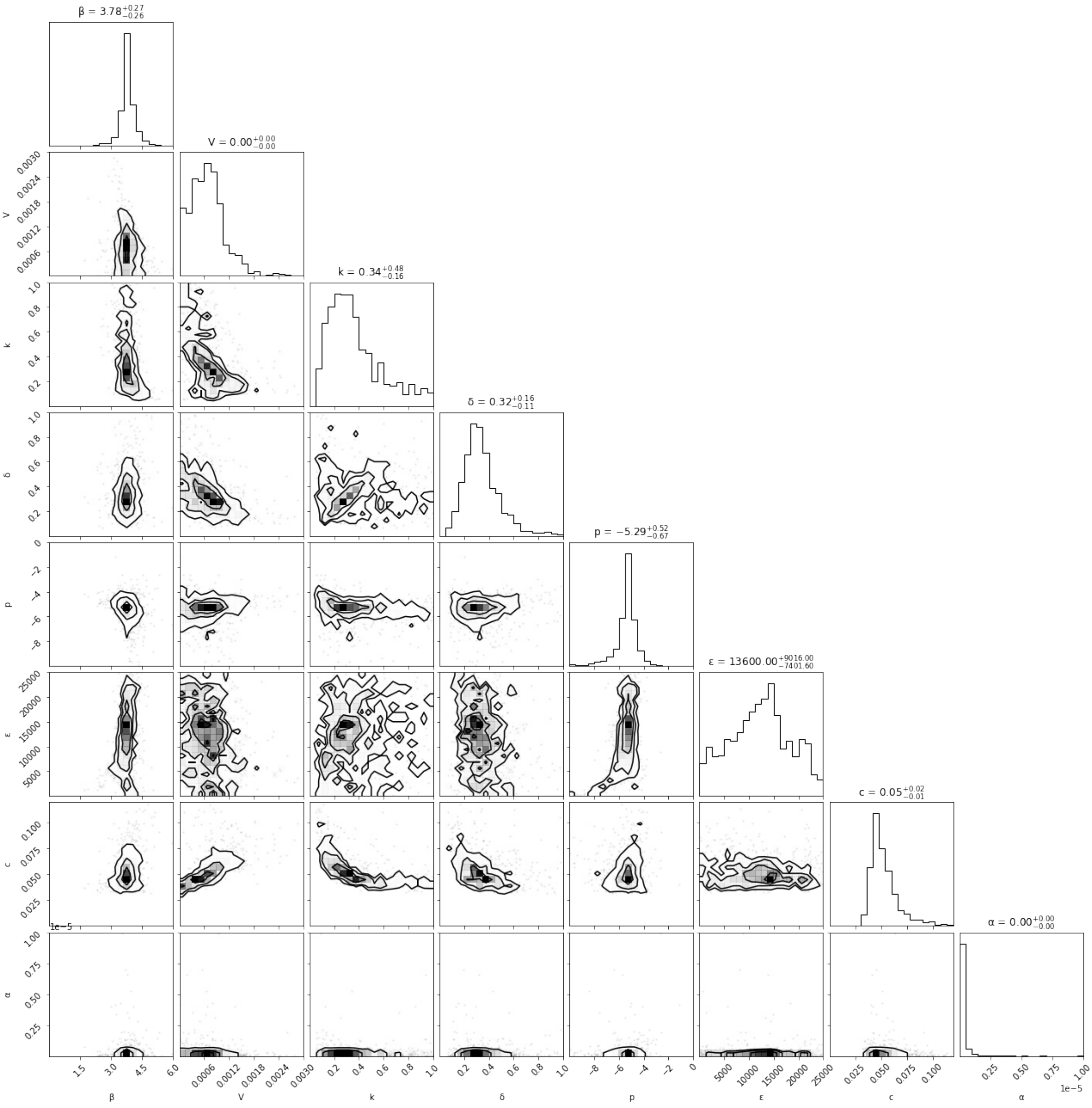


Figure 9: Ad6d24.P19 with immune response.

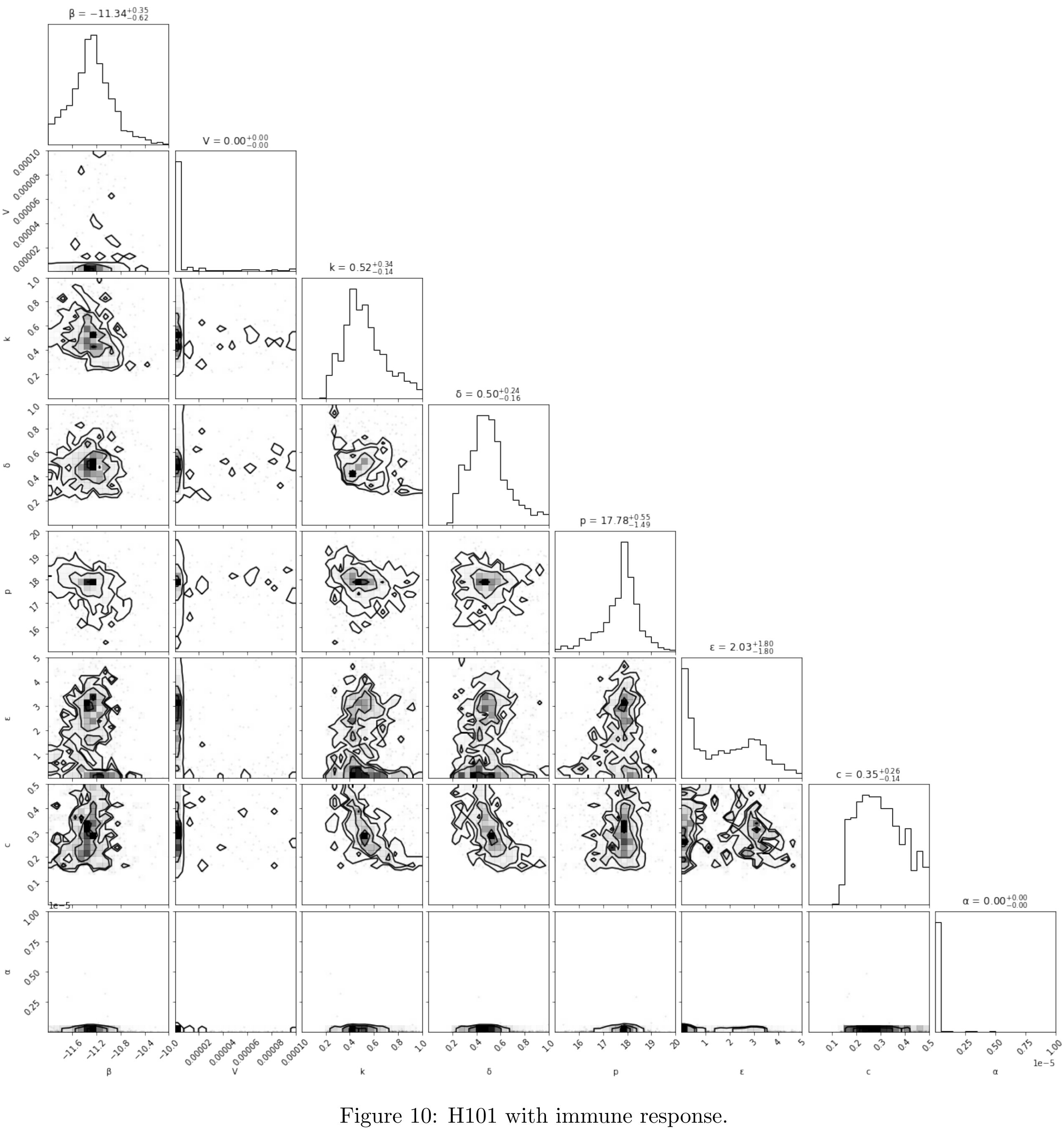


Figure 10: H101 with immune response.

## 2.1 Mann-Whitney Test tables

The following tables give p-values for the pair-wise comparisons of parameter estimates for different viral strains. p values less than 0.05 are considered significant and are shown in bold.

Table 1: p-values of $\beta$

| | Ad2d24.P19 | Ad5.P19 | Ad6d24.P19 | H101 |
|---|---|---|---|---|
| Ad1d24.P19 | **0.03** | 0.46 | **0.01** | **0.0002** |
| Ad2d24.P19 | | **0.001** | 0.41 | **0.0002** |
| Ad5.P19 | | | **0.0008** | **0.0002** |
| Ad6d24.P19 | | | | **0.0002** |

Table 2: p-values of $V$

| | Ad2d24.P19 | Ad5.P19 | Ad6d24.P19 | H101 |
|---|---|---|---|---|
| Ad1d24.P19 | **0.03** | 0.30 | **0.03** | **0.02** |
| Ad2d24.P19 | | **0.0002** | 0.42 | 0.09 |
| Ad5.P19 | | | **0.0002** | **0.0003** |
| Ad6d24.P19 | | | | **0.03** |

Table 3: p-values of $k$

| | Ad2d24.P19 | Ad5.P19 | Ad6d24.P19 | H101 |
|---|---|---|---|---|
| Ad1d24.P19 | 0.12 | **0.01** | **0.04** | **0.002** |
| Ad2d24.P19 | | 0.25 | 0.38 | 0.06 |
| Ad5d24d.P19 | | | 0.49 | 0.29 |
| Ad6d24.P19 | | | | 0.26 |

Table 4: p-values of $\delta$

| | Ad2d24.P19 | Ad5.P19 | Ad6d24.P19 | H101 |
|---|---|---|---|---|
| Ad1d24.P19 | **0.0002** | **0.0002** | **0.0002** | **0.0002** |
| Ad2d24.P19 | | 0.30 | 0.25 | **0.01** |
| Ad5d24.P19 | | | 0.51 | 0.15 |
| Ad6d24.P19 | | | | 0.11 |

Table 5: p-values of $p$

| | Ad2d24.P19 | Ad5.P19 | Ad6d24.P19 | H101 |
|---|---|---|---|---|
| Ad1d24.P19 | **0.0002** | **0.0002** | **0.0004** | **0.0002** |
| Ad2d24.P19 | | **0.0009** | **0.01** | **0.0002** |
| Ad5d24.P19 | | | **0.002** | **0.0002** |
| Ad6d24.P19 | | | | **0.0002** |

Table 6: p-values of $\epsilon$

| | Ad2d24.P19 | Ad5.P19 | Ad6d24.P19 | H101 |
|---|---|---|---|---|
| Ad1d24.P19 | **0.0002** | 0.40 | **0.0002** | **0.0002** |
| Ad2d24.P19 | | **0.0002** | **0.0003** | **0.0002** |
| Ad5d24.P19 | | | **0.0002** | **0.0006** |
| Ad6d24.P19 | | | | **0.0002** |

Table 7: p-values of $c$

| | Ad2d24.P19 | Ad5.P19 | Ad6d24.P19 | H101 |
|---|---|---|---|---|
| Ad1d24.P19 | **0.0005** | **0.0003** | **0.001** | **0.0002** |
| Ad2d24.P19 | | **0.0005** | 0.05 | **0.0002** |
| Ad5d24.P19 | | | **0.0002** | 0.22 |
| Ad6d24.P19 | | | | **0.0002** |

Table 8: p-values of $\alpha$

| | Ad2d24.P19 | Ad5.P19 | Ad6d24.P19 | H101 |
|---|---|---|---|---|
| Ad1d24.P19 | **0.001** | 0.06 | **0.001** | **0.002** |
| Ad2d24.P19 | | **0.001** | 0.50 | **0.004** |
| Ad5d24.P19 | | | **0.002** | **0.002** |
| Ad6d24.P19 | | | | 0.05 |

# References


[1] Odo Diekmann, JAP Heesterbeek, and Michael G Roberts. The construction of next-generation matrices for compartmental epidemic models. *Journal of the royal society interface*, 7(47):873–885, 2010.

[2] Daniel Foreman-Mackey. corner.py: Scatterplot matrices in python. *The Journal of Open Source Software*, 1(2):24, jun 2016.
